# Mechanochemistry Reaches-Out Sensing: Peroxidase-Mimic Fe-BTC MOF for Hydrogen Peroxide Detection by a Solvent-Free Synthesis

**DOI:** 10.1021/acsomega.5c02865

**Published:** 2025-06-04

**Authors:** Giada Mannias, Alessandra Scano, Cristiana Cabriolu, Franca Sini, Sarah Hudson, Guido Ennas

**Affiliations:** † Department of Chemical and Geological Sciences, 3111 University of Cagliari, SS 554 Bivio Per Sestu, 09042 Monserrato (CA), Italy; ‡ Department of Chemical Sciences, SSPC, the Science Foundation Ireland Research Centre for Pharmaceuticals, Bernal Institute, 8808 University of Limerick, V94 T9PX Limerick, Ireland; § National Interuniversity Consortium of Materials Science and Technology (INSTM), Via Giuseppe Giusti 9, 50121 Firenze, Italy

## Abstract

A low-cost enzyme-mimic iron­(III) trimesate MOF, prepared by a novel and green mechanochemical synthetic protocol, is used for the excellent colorimetric detection of hydrogen peroxide (H_2_O_2_). The developed iron­(III) trimesate MOF, solvent-free mechanochemically synthesized under mild conditions and in a very short time (from 6 to 60 min), is an exciting potential candidate for H_2_O_2_ detection, avoiding the immobilization of additional peroxidase enzymes. The influence of pH, temperature, catalyst concentration, and incubation time on iron­(III) trimesate MOF peroxidase-mimic activity was investigated, showing a lower limit of detection (0.24 μM) and a wider linear range (7.5–75 μM) than other peroxidase-like inorganic materials typically used for colorimetric sensing. The superior catalytic properties of our material with respect to the current literature can be ascribed to the presence of defects in the MOF structure due to the milling synthesis process.

## Graphical abstract

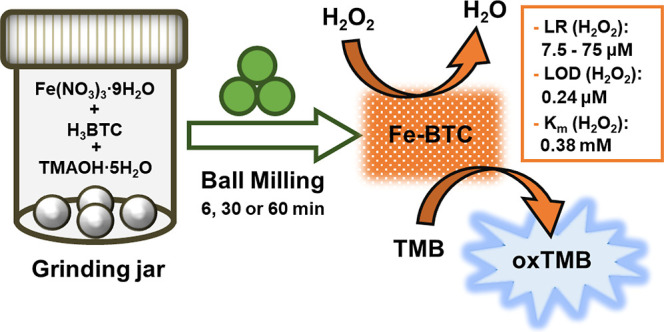

## 1 Introduction

Hydrogen peroxide (H_2_O_2_), also known as dioxygenane, is a reactive oxidizing species with disinfectant, antiviral, and antibacterial activities. Naturally produced in cells during metabolism through dismutation of superoxide anion radicals,^1^ it boasts a regulatory function in cellular signaling, including immune response, host defense, and pathogen invasion mechanisms.^2^ Due to the aforementioned antimicrobial properties, H_2_O_2_ is also widely employed at low concentrations (3–9 vol %) for medicinal applications.^3^ Its bleaching effects make it very useful in higher concentrations in the textile, paper, and food industries.^4^ But its same strong oxidizer feature makes H_2_O_2_ harmful when accumulated in the human body, inducing headaches, diabetes, and cancer as well as cardiovascular disease.^5,6^ Therefore, a practical challenge is the question of whether H_2_O_2_ is safe or toxic for living beings. The answer is in its concentration. Consequently, H_2_O_2_ detection and quantification become of great importance. Several methods are available for this purpose, which use different detection principles such as titration, chromatography, electrochemistry, or light detection depending on the application area, but all of them have a certain number of limitations. Titration and chromatography are low-cost detection methods, offering high precision. However, the titration method suffers from low efficiency and poor sensitivity,^7^ while the complicated operation procedure and derivatization in chromatography restrict its further application.^8,9^ Recently, electrochemical sensors have gained importance by allowing simple operation, short response time, and high sensitivity. Despite the noteworthy advantages, electrochemical sensors show poor specificity.^10^ In recent years, optical sensors, especially colorimetric types, have also become very popular and widespread. These sensors provide color change when influenced by external stimuli. Unlike electrochemical sensors, they possess higher accessibility, lower cost, and increasingly sensitive and selective responses toward various analytes.^11^ In particular, the use of enzymes confers to colorimetric sensors high efficiency and specificity. As an example, peroxidase enzymes [i.e., horseradish peroxidase (HRP)] in colorimetric H_2_O_2_ sensing have attracted considerable interest. HRP combines with H_2_O_2_, and the resultant complex allows oxidizing a wide range of hydrogen donors,^12^ and can be applied for the diagnosis and detection of H_2_O_2_, glucose, and ascorbic acid. The peroxidase reaction can be summarized as follows

1 $$2RH+H_{2}O_{2}\to 2R^{\cdot }+{2H}_{2}O$$

However, enzymes suffer from low stability, and their activity is affected by the external environment: temperature and pH conditions in primis. Moreover, they require high-cost and time-consuming preparation and purification, as well as special storage conditions.^13–20^ Therefore, HRP-mimic inorganic analogues have recently attracted considerable interest from researchers.^21–25^ Materials such as Fe_3_O_4_, Au or ceria nanoparticles, graphene oxide, carbon nanotubes, and carbon nanodots have been reported to exhibit catalytic activity similar to that of natural HRP.^26–31^

In this regard, Cu­(II)^32^ and Fe­(III)^33^-based metal organic frameworks, MOFs—also known as nanoenzymes^34–36^ —have recently exhibited high intrinsic peroxidase-like activity in colorimetric biosensing for H_2_O_2_.^37,38^ MOFs, in fact, as porous compounds including ion-binding selected organic ligands, not only overcome the enzyme drawbacks reported above but also have various features, such as highly porous frameworks with varied pore sizes, different functional groups, low bulk densities, and abilities to capture substances in both chemisorption and physisorption phases. Moreover, due to their high tailorability, MOFs can also provide low toxicity, excellent water stability, good biodegradability, and biocompatibility.^39^

In this work, we report on the exceptional performance in terms of linear range and better affinity toward the target substrate compared to HRP of an iron­(III) trimesate MOF. Unlike MOFs for H_2_O_2_ sensing reported in the literature,^40,41^ our MOF was synthesized via a mechanochemical approach instead of a conventional hydrothermal or solvothermal method. These methods, in fact, allow the preparation of high-quality single crystals, but working at a very low pH (pH < 1) and owing to the need for a large amount of solvent as well as a high energy input to reach the boiling point of the same solvent; thus, time- and energy-consuming synthetic methods (a more detailed comparison is reported in Table S1). Mechanosynthesis, on the contrary, allows green, biocompatible synthesis conditions without additional solvents.^42–45^ During the past decade, mechanochemistry has been extensively used for the preparation of different types of MOFs with a high degree of versatility.^46–48^ In particular, Friščić reported on the thermal treatment of discrete monomeric metal complexes, initially synthesized via mechanochemistry, which resulted in subsequent 1D or 3D metal–organic materials due to the heat treatment processes without using any solvent.^49^

Here, we present an iron­(III) trimesate MOF showing much better performance with respect to analogous peroxidase mimics reported in the literature,^4,40,41,50–52^ which was prepared by mechanochemistry, working at room temperature (RT) and for a very short time—between 6 and 60 min. This is especially mandatory to move from academic studies to industrial production,^53–55^ which requires safe, sustainable manufacturing routes with low environmental impact and high energy efficiency, according to “The Green Chemistry Principles”,^56^ in an era when alternative and greener options urge to be addressed in every field of human life.

## 2 Experimental Section

### 2.1 Chemicals and Materials

1,3,5-Benzenetricarboxylic acid (H_3_BTC, 95%), iron­(III) nitrate nonahydrate (Fe­(NO_3_)_3_·9H_2_O, 98%), tetramethylammonium hydroxide pentahydrate (TMAOH·5H_2_O, 97%), 3,3′,5,5′-tetramethylbenzidine (TMB, ≥99%), H_2_O_2_ solution (H_2_O_2_, 30% (w/w) in H_2_O), HRP (Type VI-A, 950–2000 units/mg solid (using ABTS) EC 1.11.1.7), ethanol (EtOH, ≥99.8%), sodium acetate trihydrate (NaAc·3H_2_O), and glacial acetic acid (AcOH, ≥99%) were purchased from Sigma-Aldrich. Ultrapure water (18.2 MΩ·cm) was obtained from a Millipore Milli-Q water purification system. All chemicals were used as received without any further purification.

### 2.2 Characterization

Attenuated total reflection–Fourier transform infrared (ATR–FTIR) spectra were collected on a Bruker Tensor 27 spectrophotometer (scanning range: 400–4000 cm^–1^). X-ray powder diffraction (XRPD) patterns were recorded with Cu Kα radiation (λ = 1.54056 Å) using a Bruker D8 Advance Diffractometer (scanning range: 7–80° 2θ; step size: 0.05° 2θ; *V* = 40 kV; *I* = 30 mA). Due to the high iron fluorescence emission stimulated by the Cu Kα radiation, an appropriate acquisition time was selected to obtain a satisfactory signal-to-noise ratio in the XRPD pattern.

ATR-FTIR spectra were collected on a Bruker Tensor 27 spectrophotometer (scanning range: 400–4000 cm^–1^).

Morphological analysis was carried out using transmission electron microscopy (TEM, JEOL JEM 1400 Plus electron apparatus equipped with a CCD camera at an acceleration voltage of 80 kV) and scanning electron microscopy (SEM, FEI Quanta 200 microscope).

N_2_ physisorption experiments were carried out on a Sorptomatic 1990 CE apparatus (Fisons Instruments) at −196 °C. Before the measurement, samples were degassed at 150 °C under a vacuum for 17 h. Specific surface area (SSA) and pore size distribution (PSD) were estimated from adsorption data by applying the Dubinin–Radushkevich (DR) equation and the Horvath–Kavazoe (HK) method,^57,58^ respectively.

Thermogravimetric analysis (TGA) and differential scanning calorimetry analysis (DSC) were performed under an O_2_ flow (40 mL/min) using a PerkinElmer STA6000 simultaneous thermal analyzer (temperature range: 25–850 °C; heating rate: 10 °C/min; alumina sample pan).

Ultraviolet–visible (UV–vis) spectroscopy measurements were recorded at 652 nm by using a BioTek Synergy H1 Plate Reader.

### 2.3 Synthesis of Fe-BTC

0.47 g of H_3_BTC, 1.29 g of Fe­(NO_3_)_3_·9H_2_O, and 1.81 g of TMAOH·5H_2_O were placed in a 32.0 mL stainless-steel Teflon-coated grinding jar with 13.7 g of zirconia balls. No additional solvents were added. The reaction mixture was ground using a Spex 8000 Mixer/Mill for 6, 30, or 60 min. The resulting dense, orange slurry was dispersed in 20 mL of Milli-Q deionized water. The pH of the obtained dispersion was ca. 4.0. The solid was recovered by centrifugation (2500 rpm; 10 min), washed twice with Milli-Q water, and air-dried at RT.

### 2.4 Intrinsic Peroxidase-like Activity of Fe-BTC

Enzyme-mimic activity of Fe-BTC was explored by examining four different reaction systems in acetate buffer (10 mM, pH = 4): (i) TMB (0.4 mM); (ii) TMB (0.4 mM) and Fe-BTC (250 μg/mL); (iii) TMB (0.4 mM) and H_2_O_2_ (0.5 mM); (iv) TMB (0.4 mM), Fe-BTC (250 μg/mL), and H_2_O_2_ (0.5 mM). Solution mixtures were incubated under magnetic stirring (150 rpm) at 35 °C for 1 h. Then, test samples were centrifuged (5000 rpm; 10 min), and supernatants were collected. Absorbance spectra of the supernatants were recorded in the 500–800 nm range.

### 2.5 Effect of pH, Temperature, Concentration, and Incubation Time on Fe-BTC Peroxidase-Mimic Activity

The intrinsic peroxidase-mimic activity of Fe-BTC was evaluated over the pH range of 3–5. The influence of temperature was examined from 25 to 55 °C, and different incubation times (30, 60, and 120 min) were investigated. The effect of Fe-BTC concentration (250, 500, or 800 μg/mL) on its peroxidase-mimic activity was also considered. A typical experiment was carried out by incubating the reaction mixture (Fe-BTC (250 μg/mL) or HRP (0.2 ng/mL), TMB (0.4 mM), and H_2_O_2_ (0.5 mM) in acetate buffer (10 mM, pH = 4)) at 35 °C under magnetic stirring (150 rpm) for 1 h, unless otherwise stated. Then, test samples were centrifuged (5000 rpm; 10 min), and supernatants were collected. Absorbance of the supernatants was recorded at 652 nm. All measurements were performed in triplicate, and the average value was reported. The relative activity among samples was calculated using the following formula^59^

2 $$relative\;activity\;\left(\%\right)=\frac{A-A_{0}}{A_{\max}-A_{0}}\times 100\%$$

where *A* is the absorbance recorded for each sample, *A*_0_ is the absorbance of the blank sample (H_2_O_2_-free sample), and *A*_max_ is the maximum absorbance value recorded among a set of samples.

#### 2.5.1 Steady-State Kinetic Assay

*K*_m_ and *V*_max_ (maximum reaction rate) of Fe-BTC and HRP were estimated by the Michaelis–Menten equation^60^

3 $$V_{0}=\frac{V_{\max}\cdot \left[S\right]}{K_{m}+\left[S\right]}$$

where [*S*] is the substrate concentration—[TMB] [H_2_O_2_]—and *V*_0_ is the initial reaction rate. Moreover,

4 $$V_{0}=\frac{\Delta A}{\Delta t\cdot \epsilon }$$

where Δ*t* is the time variation, Δ*A* is the change in absorbance, and ε is the molar extinction coefficient (oxidized TMB molar extinction coefficient (ε_652nm_) = 3.9 × 10^4^ M^–1^ cm^–1^).^61^

Given the double reciprocal of the Michaelis–Menten equation

5 $$\frac{1}{V_{0}}=\frac{K_{m}}{V_{\max}}\frac{1}{\left[S\right]}+\frac{1}{V_{\max}}$$

*V*_max_ and *K*_m_ can be calculated from the intercept and the slope of the double reciprocal plot (also known as the Lineweaver–Burk plot), respectively.^60^

To this purpose, the change in absorbance at 652 nm was monitored at RT for 180 s for the following dispersions in acetate buffer (10 mM; pH = 4):

(i) Fe-BTC (250 μg/mL) or HRP (1 ng/mL), H_2_O_2_ (0.5 mM), and different concentrations of TMB (0.05–0.8 mM for Fe-BTC, or in alternative, 0.1–0.8 mM for HRP);
(ii) Fe-BTC (250 μg/mL) or HRP (1 ng/mL), TMB (0.2 mM), and different concentrations of H_2_O_2_ (0.25–6 mM).

### 2.6 Colorimetric Detection of H_2_O_2_

A dose-response curve for H_2_O_2_ detection was obtained by adding Fe-BTC (250 μg/mL), TMB (0.4 mM), and different concentrations of H_2_O_2_ (0–200 μM) in acetate buffer (10 mM, pH = 4). The solution mixtures were incubated at 35 °C under magnetic stirring (150 rpm) for 1 h. Then, the test samples were centrifuged (5000 rpm; 10 min), and supernatants were collected. Absorbance of the supernatants was recorded at 652 nm. All measurements were performed in triplicate, and the average value was reported. The limit of detection (LOD) and the limit of quantification (LOQ) for H_2_O_2_ were calculated by using the following equations^62^

6 $$LOD=\frac{3\sigma }{s}$$

7 $$LOQ=\frac{10\sigma }{s}$$

where σ represents the standard deviation of ten blank tests and *s* is the slope of the calibration curve.

## 3 Results and Discussion

### 3.1 Physical-Chemical Characterization of Fe-BTC

Samples were characterized by ATR-FTIR, XRPD, TGA, DSC, and N_2_ physisorption. The effect of grinding time on the microstructure, thermal stability, and textural properties of the material was investigated. To distinguish among different grinding times varying from 6 to 60 min, Fe-BTC samples were labeled as Fe-BTC_6 min, Fe-BTC_30 min, and Fe-BTC_60 min, respectively. XRPD patterns obtained for as-synthesized samples are depicted in Figure 1. Despite the different grinding times, there are no substantial microstructure variances among samples. Indeed, the position of diffraction peaks is similar for all samples, as well as comparable to the XRPD pattern reported in the literature for this material, attesting to the formation of a Fe-BTC framework.^63,64^ An intense, broad diffraction peak (at ca. 10.65° 2θ) is followed by broader and less intense diffraction peaks (at ca. 18.76, 23.83, 28.09, 33.36, and 42.49° 2θ). Such a broadness of diffraction peaks is consistent with the disordered nature of Fe-BTC in comparison with its crystalline counterpart (i.e., MIL-100­(Fe)).^65^

**Figure 1:**
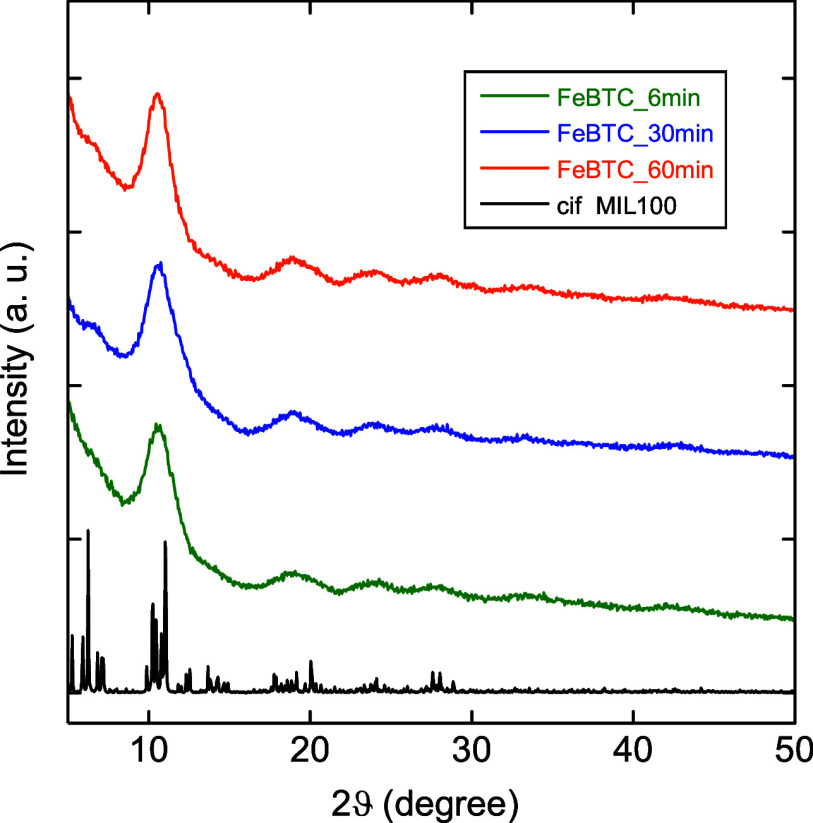
XRPD patterns of Fe-BTC_6 min, Fe-BTC_30 min, and Fe-BTC_60 min and simulated XRPD pattern of the MIL-100­(Fe) phase.

Fe-BTC_6 min, Fe-BTC_30 min, and Fe-BTC_60 min show similar ATR-FTIR spectra (Figure 2), further attesting that the different grinding time does not affect the microstructure of the MOF. The stretching vibrations deriving from OH^–^ groups and H_2_O molecules coordinated to iron trimers and adsorbed water give rise to a broad band between 3600 and 3100 cm^–1^, which is likely to mask the weak band due to aromatic C–H stretching vibrations at ca. 3080 cm^–1^.^66,67^ The weak carbonyl band (at ca. 1703 cm^–1^) is related to the extra-framework or partially deprotonated H_3_BTC molecules, which had been found to interrupt the order of the network, leading to a disordered structure.^68^ The band at ca. 1625 cm^–1^ arises from the C=O stretching of COO^–^ groups, whereas stretching vibrations of O–C–O bonds account for the bands at ca. 1564 and 1371 cm^–1^. The bands at ca. 759 and 706 cm^–1^ are attributed to the aromatic C–H bending vibrations, and the band at 463 cm^–1^ is related to the stretching of the Fe–O bond.^69–71^

**Figure 2:**
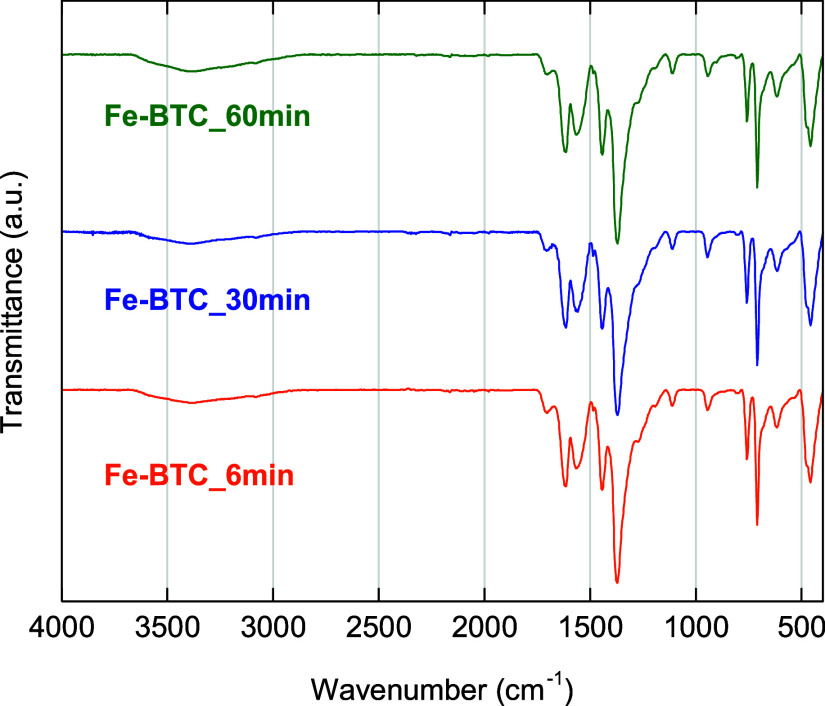
ART-FTIR spectra of Fe-BTC_6 min, Fe-BTC_30 min, and Fe-BTC_60 min.

Thermogravimetric (TG) curves, corresponding derivative curves (dTG), and DSC curves obtained for Fe-BTC_6 min, Fe-BTC_30 min, and Fe-BTC_60 min (Figure 3) reveal no significant differences concerning the thermal behavior of samples despite varying grinding time. Indeed, three weight losses are displayed for all samples (Figure 3a), consistent with TG curves reported in the literature for this material.^72,73^ The first weight loss (25–150 °C), due to the loss of adsorbed H_2_O, is slightly higher for Fe-BTC_6 min compared to Fe-BTC_30 min and Fe-BTC_60 min. However, this is not a relevant difference between samples since water adsorption depends on external variables (e.g., ambient humidity and exposure time to air).^74^ The second weight loss (150–260 °C) arises from the loss of water molecules coordinated to iron trimers. The third and last weight loss (260–330 °C) range is attributed to the collapse of the framework into hematite (XRPD Figure S3). Such a decomposition occurs by combustion, as attested to by an intense exothermic peak at ca. 340 °C in the DSC curves of the samples (Figure 3c). It is noteworthy that despite the XRPD pattern showing the formation of an amorphous material, thermogravimetric curves obtained for the 3 samples are similar to the ones obtained for an iron carboxylate MOF with a high crystallinity degree as shown in our previous study.^44^ Considering the diffraction results, we would have expected a thermal behavior like the commercial basolite F300.^44^ This could be explained by the formation of a composite material, made up of nanocrystals and an amorphous matrix, in agreement with the typical morphology of Fe-BTC reported in the literature, and visible by transmission and scanning electron microscopy analysis. Figures S1 and S2 show the presence of large particles covered with smaller nanoparticle sludge, which decreases with increasing milling time.

**Figure 3:**
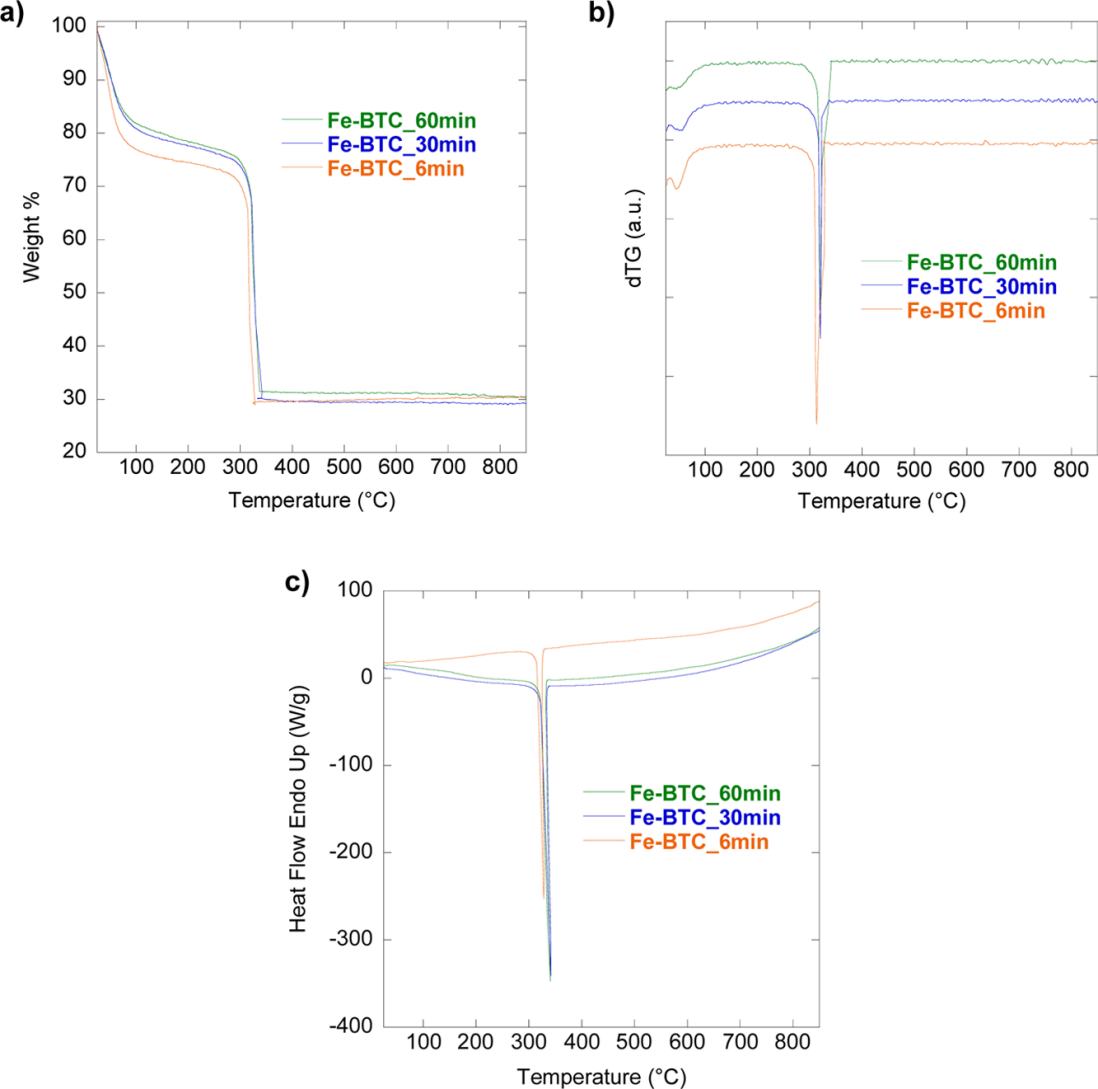
TG (a), dTG (b), and DSC (c) curves obtained for Fe-BTC_6 min, Fe-BTC_30 min, and Fe-BTC_60 min samples.

N_2_ adsorption/desorption isotherms and PSD curves for Fe-BTC_6 min, Fe-BTC_30 min, and Fe-BTC_60 min are shown in Figure 4. All samples present a I-type isotherm (Figure 4a), which is typical of microporous material,^75^ attaining a plateau at ca. *p*/*p* ^0^ = 0.12. As depicted in Figure 4b, PSD curves of all samples show a peak centered at ca. 6 Å, followed by a less intense and broader peak at ca. 12 Å, indicating the presence of two types of microporous windows, similar to MIL-100­(Fe). Such a second peak is more pronounced for Fe-BTC_60 min, indicating a slightly larger population of 12 Å-diameter pores compared to the other samples.

**Figure 4:**
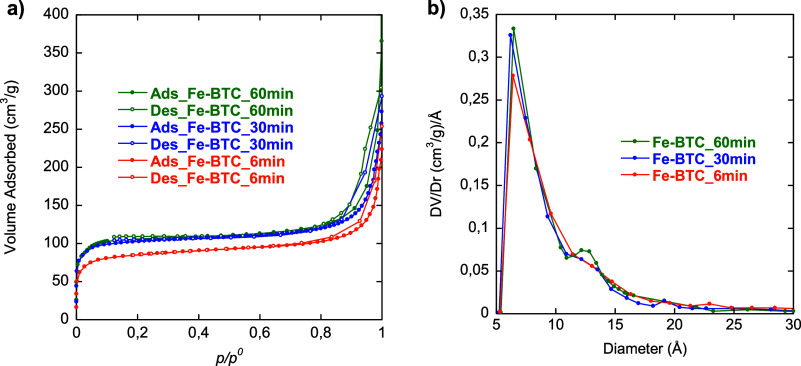
N_2_ adsorption/desorption isotherms (a) and PSD curves (b) of Fe-BTC_6 min, Fe-BTC_30 min, and Fe-BTC_60 min.

All samples are essentially microporous, with a surface area ranging from 450 to 370 m^2^/g (Table 1).

**Table 1:** SSA and Macro-, Meso-, and Micropore Volume of Fe-BTC_6 min, Fe-BTC_30 min, and Fe-BTC_60 min^a^

	Fe-BTC_60 min	Fe-BTC_30 min	Fe-BTC_6 min
SSA (m^2^/g)	450	449	370
micropore volume (cm^3^/g)	0.162	0.157	0.129

a % relative standard deviation (% RSD) SSA = 2; % RSD pore volume = 1.

Therefore, not only thermal stability and microstructure but also textural properties of this material are not considerably affected by the different grinding times. Given such physical-chemical similarities between Fe-BTC_6 min, Fe-BTC_30 min, and Fe-BTC_60 min, 6 min grinding should be preferred over longer synthesis. Indeed, time-saving procedures are more appealing to industrial scale-up owing to lowering the costs of the overall production process. Furthermore, the fast synthesis and the absence of any solvents are desirable in terms of biocompatibility and sustainability, accounting for considerably milder reaction conditions compared to hydrothermal and batch-based synthesis.^76,77^ Therefore, further investigations on the intrinsic peroxidase mimic presented in this work have been performed on Fe-BTC_6 min (hereafter, Fe-BTC).

### 3.2 Intrinsic Peroxidase-like Activity

The intrinsic enzyme-mimic behavior of the Fe-BTC MOF was evaluated by investigating its capability to catalyze the oxidation of peroxidase substrates in the presence of H_2_O_2_. 3,3′,5,5′-Tetramethylbenzidine (TMB) has been selected as a chromogenic peroxidase substrate owing to providing higher detection sensitivity than other dyes used for sensing purposes (e.g., 2,2′-azino-bis­(3-ethylbenzothiazoline-6-sulfonic acid) diammonium salt (ABTS) and *o*-phenylenediamine (OPD)).^78^ Peroxidase mimic activity of Fe-BTC originates from its catalytic activation of H_2_O_2_ through electron transfer, which leads to ^•^OH radicals by a Fenton-like reaction involving Fe^3+^ ions and H_2_O_2_, as shown in eqs 8–11 (Scheme 1)^40,78^

8 $$Fe^{3+}+H_{2}O_{2}⇄Fe\cdot \cdot \cdot OOH^{2+}+H^{+\cdot }$$

9 $$Fe\cdot \cdot \cdot OOH^{2+}\to HOO^{\cdot }+Fe^{2+}$$

10 Fe2++H2O2→OH·+OH−+Fe3+

11 OH·+RH→R·+H2O

**Scheme 1:**
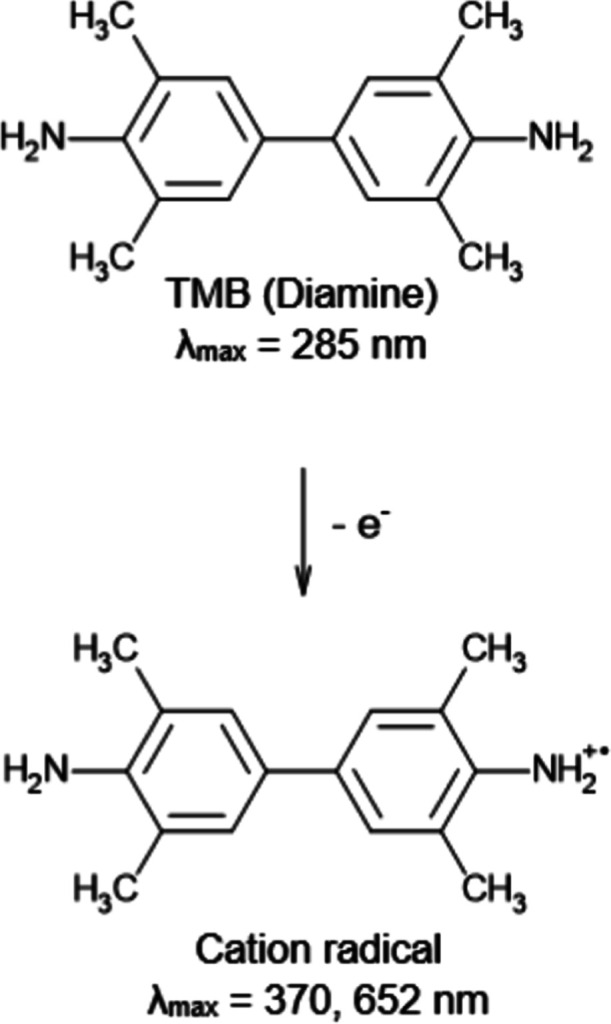
Oxidation of 3,3′,5,5′-Tetramethylbenzidine (TMB) Catalyzed by Fe-BTC

**Scheme 2:**
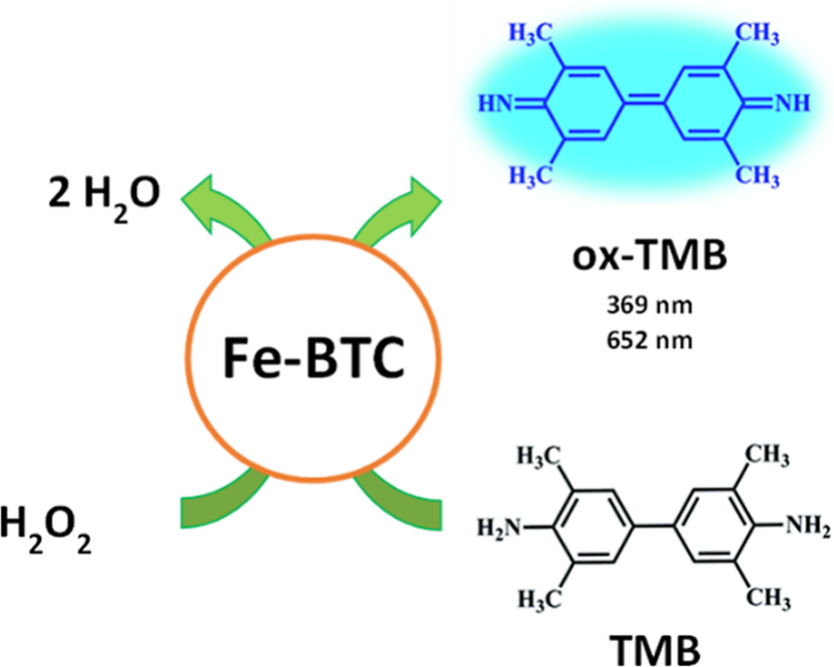
Schematic Representation of the Enzyme Mimic Cascade Reaction Involving Fe-BTC^a^

a In the presence of H_2_O_2_, Fe-BTC can catalyze the oxidation of TMB into its blue-colored oxidized form (ox-TMB).

The resulting ^•^OH radicals can react with chromogenic dye 3,3′,5,5′-tetramethylbenzidine (TMB), producing a color change in the reaction. In the presence of H_2_O_2_, the peroxidase-mimicking nanozyme Fe-BTC catalyzes the conversion of H_2_O_2_ into hydroxyl free radicals, which oxidize the colorless 3,3′,5,5′-tetramethylbenzidine (TMB) into a blue product (ox-TMB), Scheme 2.

As shown in Figure 5, the characteristic maximum absorbance peak of oxidized TMB (ox-TMB) at 652 nm (*A*_652nm_) is not observed when neither H_2_O_2_ nor Fe-BTC is present. A weak increase in the absorbance is detected when the reaction system contains both TMB and Fe-BTC, revealing a modest oxidation capacity of the MOF to oxidize TMB in the absence of H_2_O_2_. A slightly higher *A*_652nm_ is visible in the presence of both TMB and H_2_O_2_. However, only when the reaction system contains TMB, H_2_O_2_, and Fe-BTC, a significantly higher *A*_652nm_ is detected, attesting to the intrinsic enzyme-mimic capability shown by the as-synthesized Fe-BTC to catalyze the oxidation of peroxidase substrates in the presence of H_2_O_2_.

**Figure 5:**
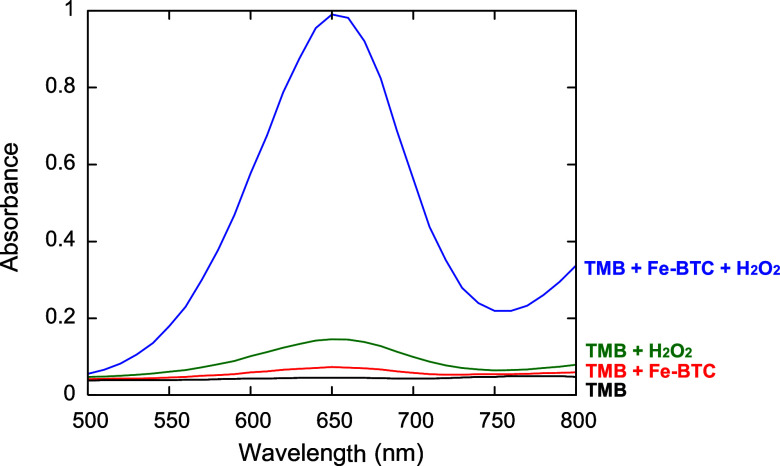
Absorption spectra of different reaction systems in acetate buffer (10 mM, pH 4), recorded after 1 h incubation at 35 °C. TMB, H_2_O_2_, and Fe-BTC (blue), TMB and H_2_O_2_ (green), TMB and Fe-BTC (red), and TMB (blue). Concentrations: 0.5 mM H_2_O_2_, 0.4 mM TMB, and 250 μg/mL Fe-BTC.

### 3.3 Activity Dependence on pH, Temperature, Incubation Time, and Concentration

Analogous to natural enzymes, enzyme mimics’ activity is strictly influenced by experimental conditions. Hence, prior to performing colorimetric detection experiments, the effect of pH, temperature, concentration, and incubation time on the intrinsic peroxidase-like activity of Fe-BTC was investigated to identify the best reaction conditions. Unlike HRP, whose optimum has been found at pH 4.5 (Figure 6b), the peroxidase-like activity of Fe-BTC gradually decreased as the pH decreased, significantly decreasing at higher pH (Figure 6a), as similarly evidenced by Zhang et al. for crystalline MIL-100­(Fe).^40^ Even though the highest activity was attained at pH 3.5 for Fe-BTC, pH 4 has been selected as the standard condition in the following activity analysis. Indeed, mild reaction conditions are preferred in terms of biocompatibility of the detection assay, being feasible to be coupled to natural enzymes for cascade reaction-based biosensing (e.g., glucose and cholesterol detection).^79,80^ Fe-BTC shows a temperature profile comparable to that of HRP (Figure 6b,c, respectively), with a maximum at 35 °C and a sharp activity loss above 45 °C. The effect of incubation time was also investigated, reaching the optimum after 60 min (Figure 6e). As shown in Figure 6f, activity decreases with increasing concentrations of Fe-BTC. This could be explained by considering that higher concentrations of Fe-BTC provide a larger surface available to adsorb and remove oxTMB from solution, becoming undetectable by UV–vis spectroscopy. Indeed, the solid collected by centrifugation after 1 h of incubation is blue-colored, attesting to the adsorption of oxTMB on Fe-BTC. Moreover, the amount of such a blue solid increases with increasing Fe-BTC concentrations (Figure 7).

**Figure 6:**
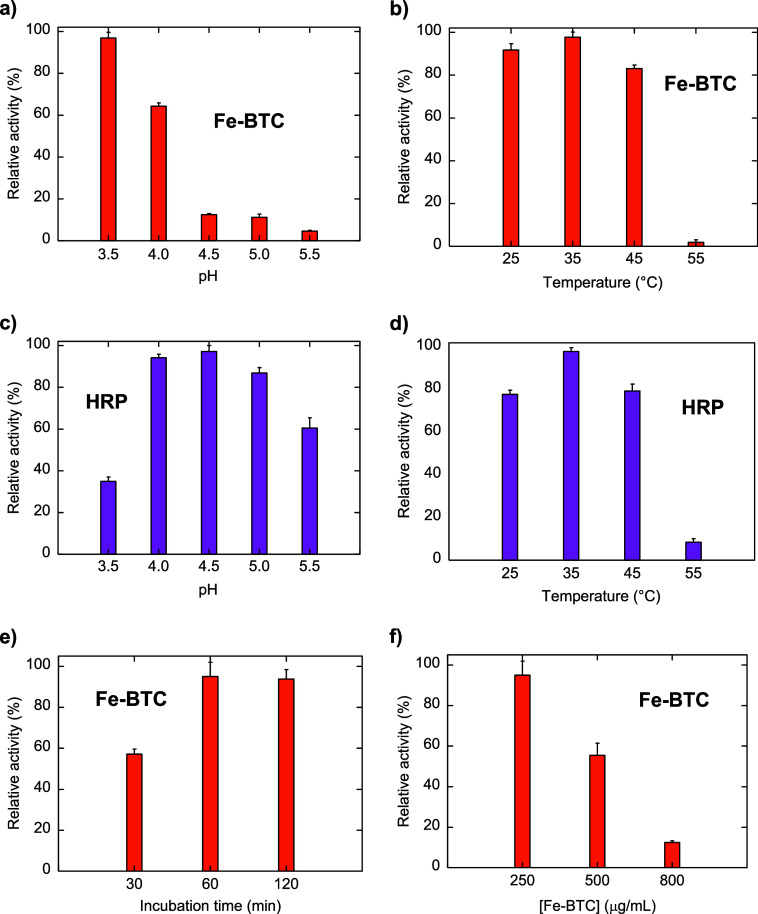
Intrinsic peroxidase-like activity of Fe-BTC depends on pH (a), temperature (b), incubation time (e), and concentration (f). pH (c) and temperature (d) profiles of HRP under the same reaction conditions are given for comparison. Error bars represent the standard deviations of three independent experiments.

**Figure 7:**
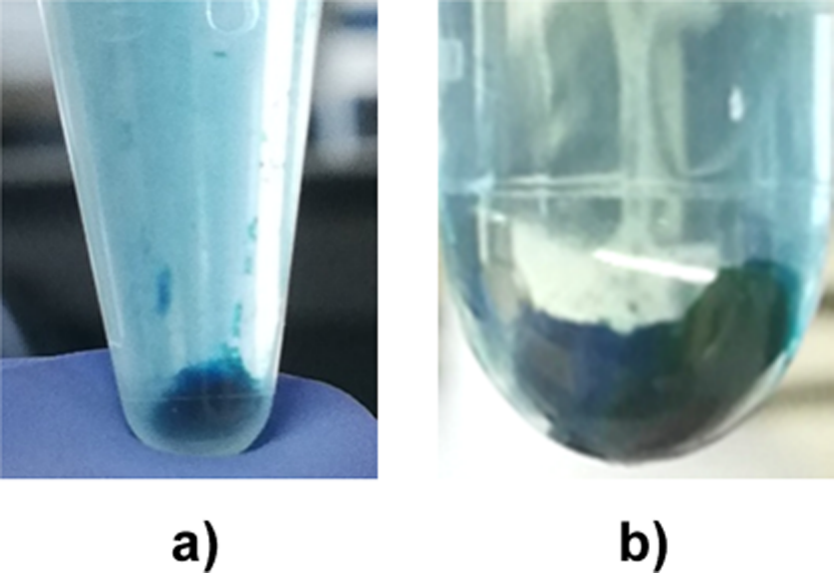
Blue-colored solids collected by centrifugation of reaction mixtures with different Fe-BTC concentrations: Fe-BTC 250 μg/mL (a) and Fe-BTC 800 μg/mL (b).

According to the results discussed above, the optimal pH, temperature, incubation time, and Fe-BTC concentration to perform the H_2_O_2_ colorimetric detection assay are pH = 4.0, 35 °C, 60 min, and 250 μg/mL, respectively.

### 3.4 Steady-State Kinetic Assay

Kinetic studies have been carried out to further investigate the intrinsic peroxidase-like activity of Fe-BTC. Figure 8 shows Michaelis–Menten curves and Lineweaver–Burk double-reciprocal plots obtained for both Fe-BTC and HRP. As resumed in Table 2, *K*_m_ (TMB substrate) for Fe-BTC is slightly higher than HRP, attesting to a relatively lower affinity toward TMB for Fe-BTC compared to HRP. In addition, *V*_max_ (TMB substrate) for Fe-BTC is lower than the natural peroxidase, revealing a slower reaction rate for the enzyme mimic. Even though *V*_max_ (H_2_O_2_ substrate) for Fe-BTC is also lower compared to HRP, *K*_m_ (H_2_O_2_ substrate) for Fe-BTC is ca. 2.8 times lower compared to HRP, revealing a much better affinity toward H_2_O_2_ for Fe-BTC compared to the natural enzyme HRP.

**Figure 8:**
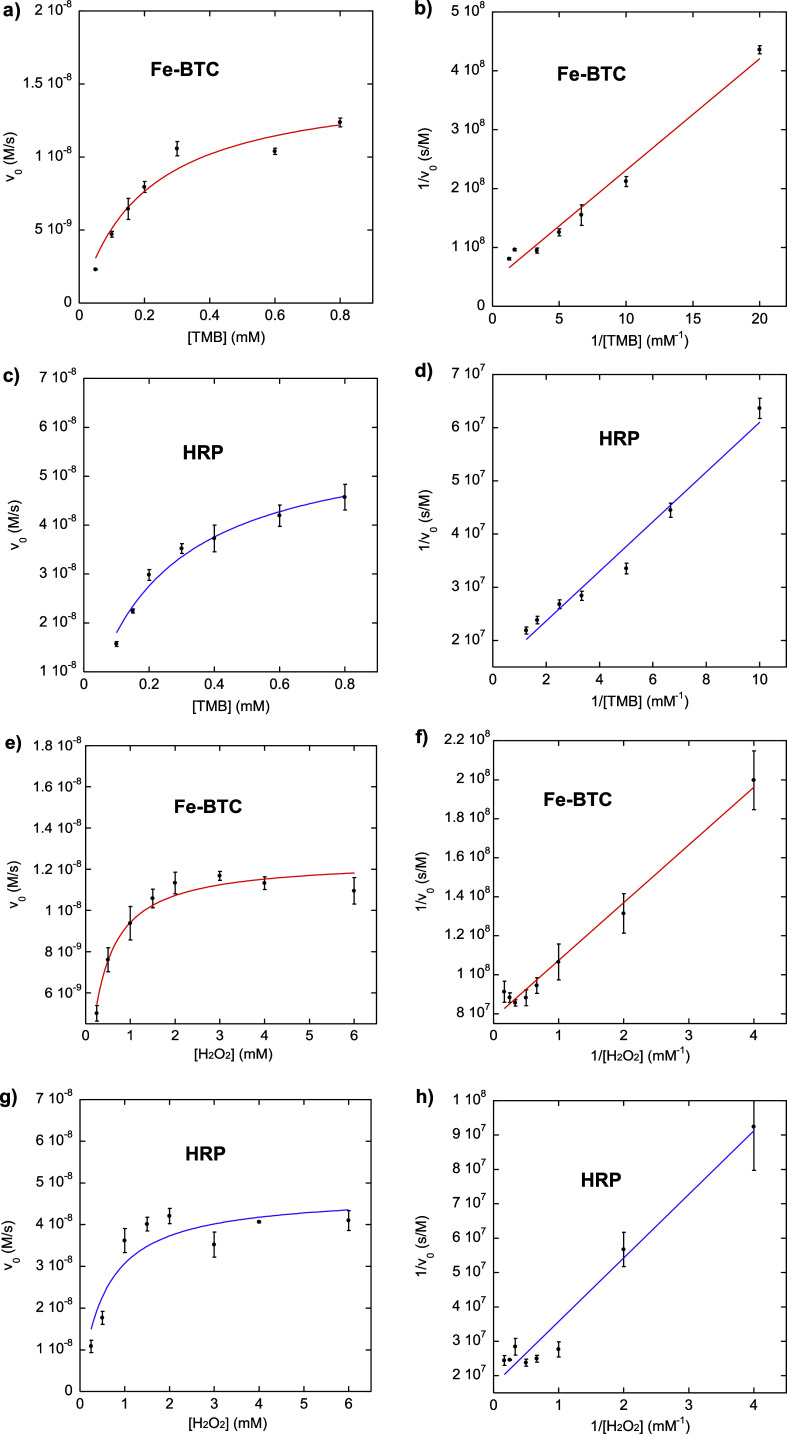
Michaelis–Menten curves obtained for Fe-BTC (a,e) and HRP (c,g) and the corresponding double-reciprocal plots for Fe-BTC (b,f) and HRP (d,h). Error bars represent the standard deviations of three independent experiments.

**Table 2:** Comparison of the Kinetic Parameters *K*_m_ and *V*_max_ Obtained for Fe-BTC and HRP^a^

catalyst	substrate	*E* (M)	*K*_m_ (mM)	*V*_max_ (M/s)
Fe-BTC	TMB	2.85 × 10^–4^	0.45	2.38 × 10^–8^
HRP	TMB	2.27 × 10^–11^	0.32	6.95 × 10^–8^
Fe-BTC	H_2_O_2_	2.85 × 10^–4^	0.38	1.29 × 10^–8^
HRP	H_2_O_2_	2.27 × 10^–11^	1.07	5.80 × 10^–8^

a “E” denotes the catalyst’s concentration. Molar weight (Fe-BTC): 876 g/mol.^81^

### 3.5 H_2_O_2_ Colorimetric Sensing

Because of the remarkable affinity toward H_2_O_2_ shown by Fe-BTC, the as-synthesized enzyme-mimic has been employed under the above-mentioned optimal conditions for the colorimetric detection of H_2_O_2_. A dose–response curve (Figure 9) shows a progressive increase of *A*_652nm_ with the increase of H_2_O_2_ concentration in a 0–200 μM [H_2_O_2_] range. A good linear correlation between *A*_652nm_ and H_2_O_2_ is found in the range 7.5–75 μM [H_2_O_2_] (*R* = 0.99961). LOD and LOQ correspond to 0.24 and 0.79 μM, respectively (σ = 0.00073787; *s* = 0.0093126).

**Figure 9:**
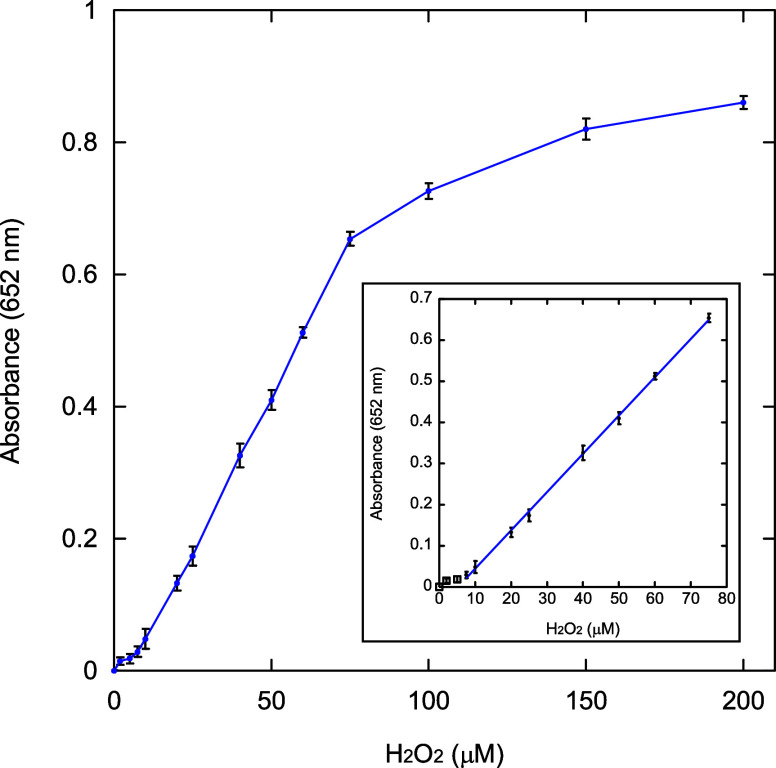
Dose–response curve between *A*_652nm_ and [H_2_O_2_] in the 0–200 μM range. Inset: linear calibration plot for H_2_O_2_ detection. Error bars represent the standard deviations of three independent experiments.

Table 3 shows a comparison of the characteristics of various peroxidase-mimic materials used for the colorimetric detection of H_2_O_2_ from the literature. The LOD calculated for the as-synthesized Fe-BTC is significantly lower compared to other peroxidase mimics reported in the literature, attesting to a remarkable sensitivity of the colorimetric detection assay presented in this work. Noteworthily, the as-synthesized Fe-BTC shows a wider linear range compared to its crystalline counterpart MIL-100­(Fe),^40^ as well as a better sensing performance compared to an Fe-BTC material obtained via the hydrothermal approach.^82^ The better catalytic performance of our Fe-BTC with respect to MIL-100 or Fe-BTC prepared with the hydro-/solvothermal approach is connected to its disordered structure. In fact, when compared to MIL-100, which exhibits a crystalline structure and Lewis’s acid sites, Fe-BTC presents both Lewis and Brönsted acid sites. The latter are constituted of terminal carboxyl groups whose presence interrupts the order of the MOF network, as detected by the FTIR analysis (Figure 2). This gives rise to a MOF structure with low crystallinity, as confirmed by the XRPD analysis, but much more efficient from the catalytic point of view. The mechanochemical process, in fact, induces the formation of a higher number of defects than the hydro-solvothermal method, improving the catalytic activity of the Fe-BTC obtained.

**Table 3:** Linear Range and LOD of Various Peroxidase Mimics Employed for H_2_O_2_ Colorimetric Sensing

catalyst	substrate	linear range (μM) H_2_O_2_	LOD (μM) H_2_O_2_	ref
FeS_2_ NPs	TMB	2–80	0.91	^83^
Fe–Ag_2_S	TMB	10–150	7.82	^84^
MOF (Co/2Fe)	TMB	10–100	5	^85^
Hemin@MIL-101(Al)–NH_2_	TMB	5–200	2	^52^
MIL-53(Fe)	TMB	0.95–19	0.13	^41^
MIL-88B-Fe	TMB	10–100	0.60	^62^
MIL-88(Fe)	TMB	2–20	0.562	^50^
MIL-68(Fe)	TMB	3–40	0.256	^40^
MIL-100(Fe)	TMB	3–40	0.155	^40^
Fe-BTC	TMB	20–3000	14	^82^
Fe-BTC	TMB	7.5–75	0.24	this work

These results further point out not only the enormous potential of Fe-BTC, often underrated because of its disordered nature, but also the advantages of green synthesis methods. Indeed, it has been proven in this work that a highly sensing performing Fe-BTC MOF can be easily obtained under extremely mild reaction conditions (6 min grinding, RT, no solvents) compared to the synthesis reported in the literature for MIL-100­(Fe),^18,24^ as well as for other representative peroxidase mimics (e.g., MIL-53­(Fe), MIL-88­(Fe), and MIL-68­(Fe)),^40,41,50^ and for analogous disordered Fe-BTC materials.^74,77,82^

## 4 Conclusions

The use of enzyme-mimic-based sensors overcomes the expensive isolation and purification steps as well as the poor thermal stability and low shelf life shown by natural enzymes. However, most enzyme mimics generally require harsh, time-consuming, and solution-based synthesis, hindering their large-scale application. Here, we have successfully carried out a colorimetric detection of H_2_O_2_ exploiting the peroxidase-like activity shown by an Fe-BTC MOF synthesized via a mechanochemical approach in just 6 min at RT without additional solvents. Good sensitivity and a wide linear range have been attained, pointing out a much better affinity toward H_2_O_2_ compared to the natural enzyme HRP and even surpassing the performance of analogous peroxidase mimics reported in the literature. Such fast, green, and facile mechanosynthesis makes the development of H_2_O_2_ sensors sustainable and extremely attractive to industrial scale-up, contributing to efficiently addressing the increasingly widespread and hazardous issues—silent plague—related to food and environmental contaminations.

## Supplementary Material



## Notes

Current synthetic methods for the preparation of MOFs and additional characterization details (TEM and SEM micrographs of Fe-BTC and X-ray diffraction pattern of Fe-BTC after thermal treatment) (PDF)
