# Supplementary material for: Mechanochemistry Reaches-Out Sensing: Peroxidase-Mimic Fe-BTC MOF for Hydrogen Peroxide Detection by a Solvent-Free Synthesis
Source: ACS Omega. 2025 Jun 4;10(23):25039–50. doi: 10.1021/acsomega.5c02865 (PMC12177600; doi:10.1021/acsomega.5c02865)
Supplement: Supplementary file 1 [file ao5c02865_si_001.pdf]

SUPPORTING INFORMATION

Mechanochemistry reaches-out sensing: peroxidase-mimic Fe-BTC MOF for H_2O_2 detection by a solvent free synthesis.

Giada Mannias[1], Alessandra Scano[1,3*], Cristiana Cabriolu[1], Franca Sini[1], Sarah Hudson[2] and Guido Ennas[1,3]

[1] Department of Chemical and Geological Sciences, University of Cagliari, SS 554 Bivio Per Sestu, 09042 Monserrato (CA), Italy

[2] Department of Chemical Sciences, SSPC, the Science Foundation Ireland Research Centre for Pharmaceuticals, Bernal Institute, University of Limerick, V94 T9PX, Limerick, Ireland

[3] National Interuniversity Consortium of Materials Science and Technology (INSTM), Via Giuseppe Giusti 9, 50121 Firenze, Italy

Table 1SI. Current synthetic methods for the preparation of MOFs – advantages and disadvantages.

Synthesis method	Advantages	Disadvantages
Conventional hydrothermal/ solvothermal method	Formation of high-quality single crystals and dense structures.	Hard reaction conditions, i.e. high temperature and pressure, use of large solvent amounts, and long synthesis times (time and energy-consuming manners); large-scale production has remained a serious challenge.
Solution method	Mild reaction conditions; single crystals obtainment.	Long reaction times; difficult scale-up.
Electrochemical method	Avoiding the problem of toxic anions, derived from metal salts, abundantly introduced to the reaction systems; mild synthesis conditions; easy operations. Large-scale synthesis elaborated by scientists from BASF.	Complex mechanism; formation of by-products.
Microwave-assisted synthesis	Shorter reaction time (from a few hours to a few minutes) without deterioration of the product quality.	Difficult scale-up.
Mechanochemical method	Solid state reactions without use of solvents; mild conditions; shorter reaction time (from 1 hour to a few minutes); easy operations; easy scale-up.	Impossible to obtain single crystals.

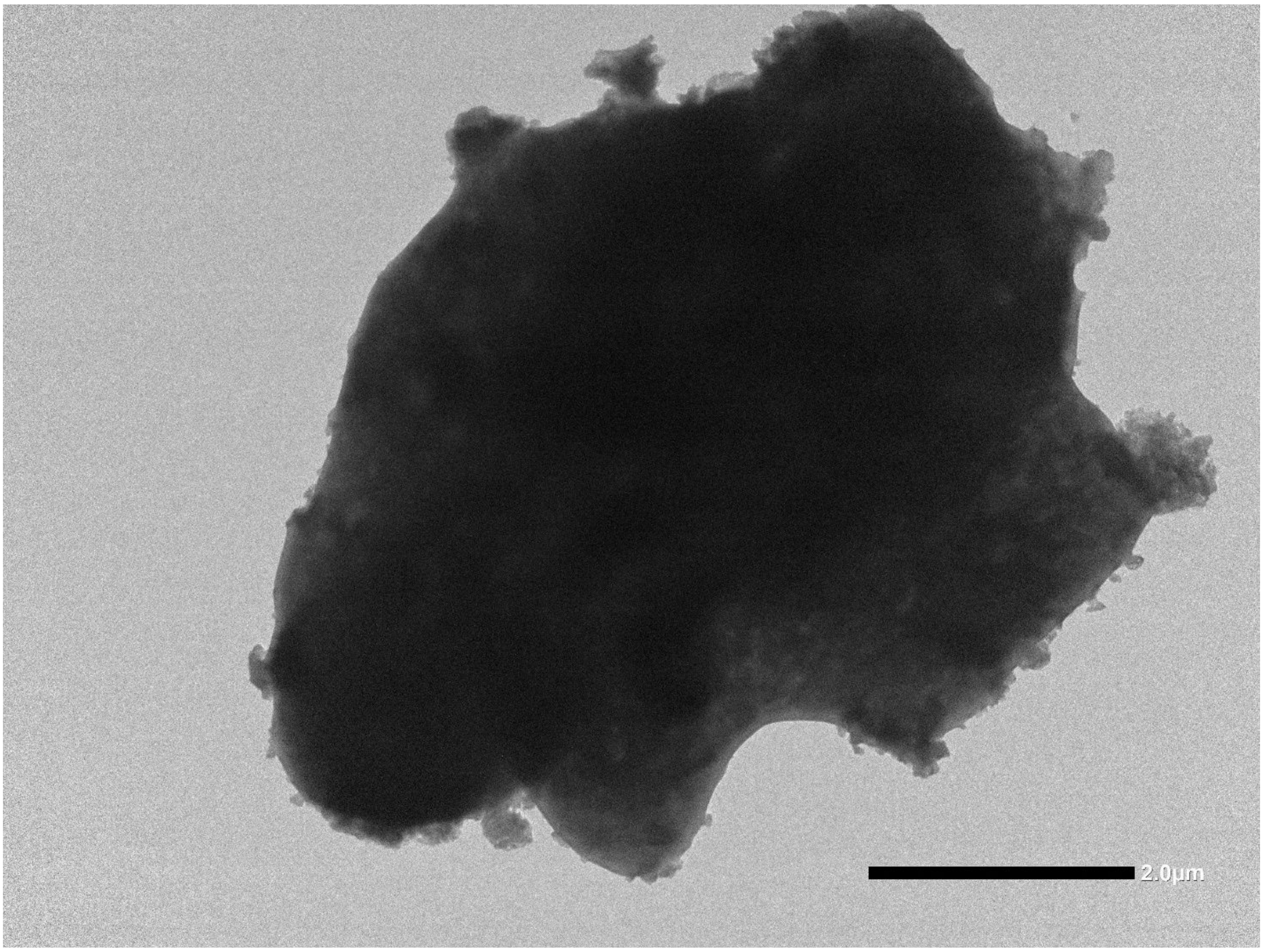

Figure 1SI. Transmission electron micrograph of Fe-BTC_60min.

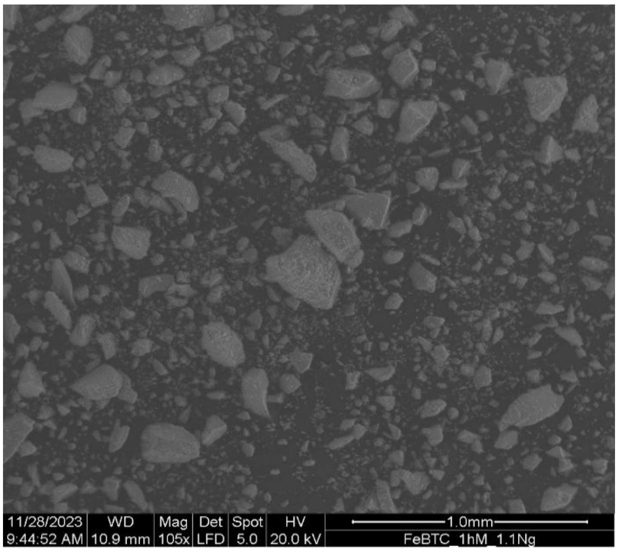
11/28/2023 9:44:52 AM
WD 10.9 mm
Mag 105x
Det LFD
Spot 5.0
HV 20.0 kV
1.0mm
FeBTC_1hM_1.1Ng

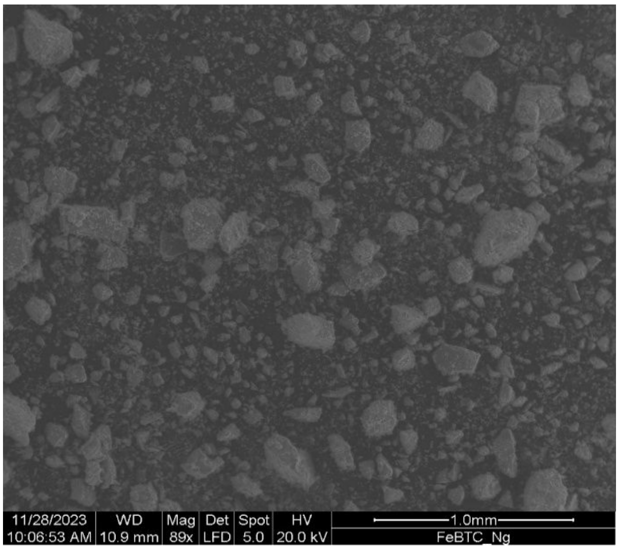
11/28/2023 10:06:53 AM
WD 10.9 mm
Mag 89x
Det LFD
Spot 5.0
HV 20.0 kV
1.0mm
FeBTC_Ng

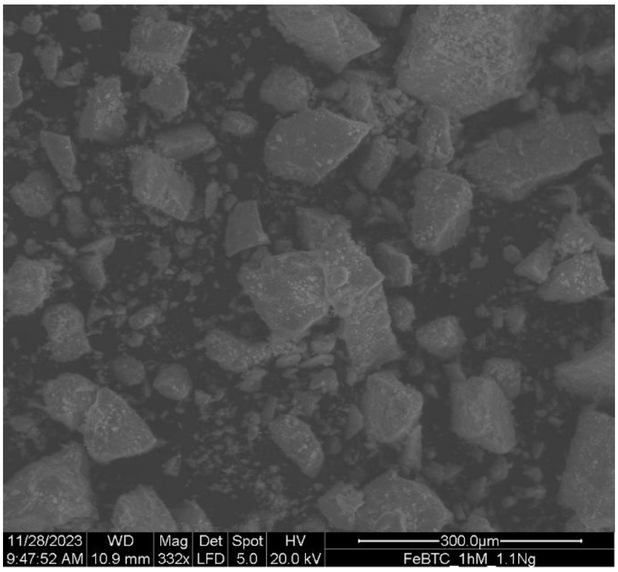
11/28/2023 9:47:52 AM
WD 10.9 mm
Mag 332x
Det LFD
Spot 5.0
HV 20.0 kV
300.0µm
FeBTC_1hM_1.1Ng

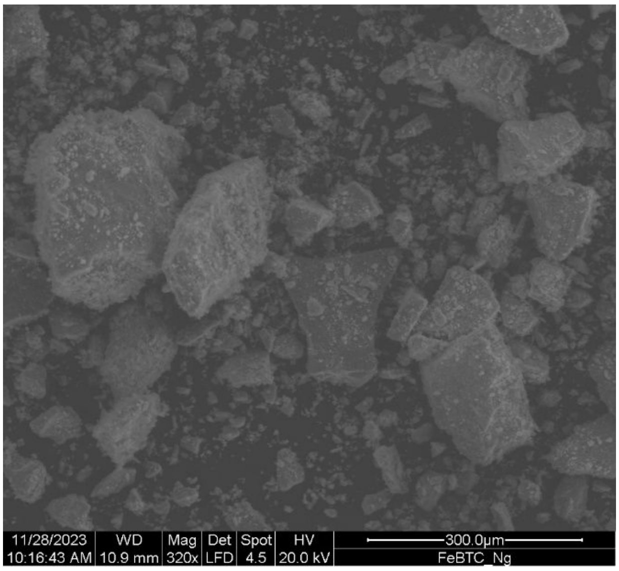
11/28/2023 10:16:43 AM
WD 10.9 mm
Mag 320x
Det LFD
Spot 4.5
HV 20.0 kV
300.0µm
FeBTC_Ng

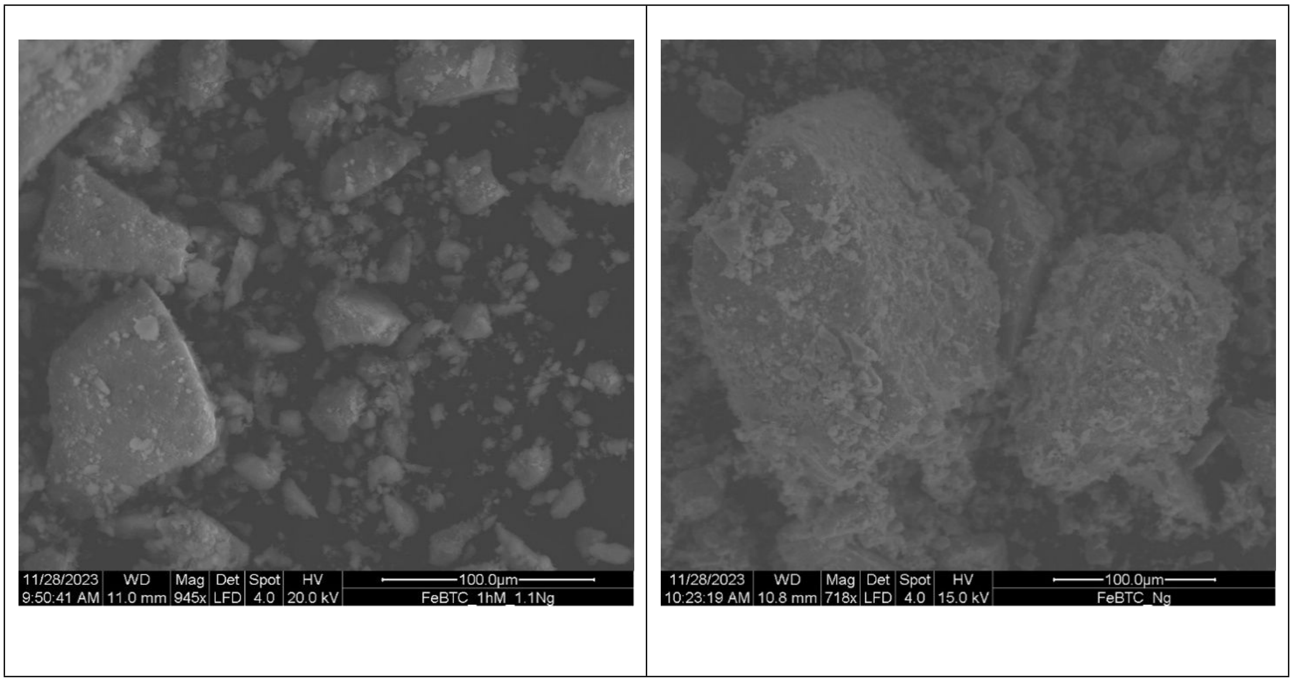

Figure 2SI. Scanning electron micrographs of Fe-BTC prepared at different milling times: Fe-BTC_60min (left side) and Fe-BTC_30min (right side). Images of the two samples are shown at different magnifications.

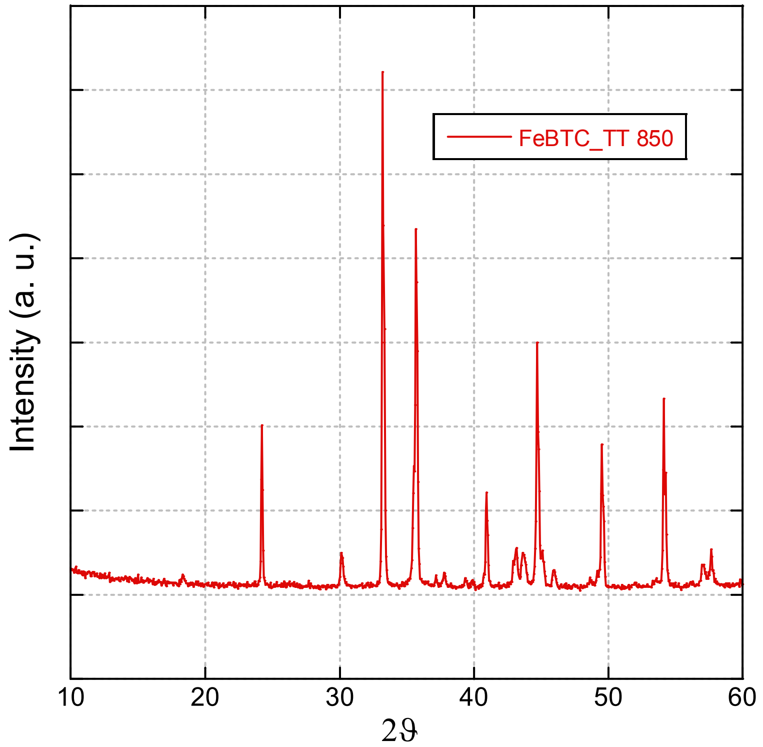

Figure 3SI. X-Ray Diffraction pattern of FeBTC_60 min after thermal treatment at 850° C.

References

[1] F. Afshariazar and A. Morsali. *The unique opportunities of mechanosynthesis in green and scalable fabrication of metal–organic frameworks*. J. Mater. Chem. A, **2022**,10, 15332-15369

[2] Z. Han, Y. Yang, J. Rushlow, J. Huo, Z. Liu, Y-C. Hsu, R. Yin, M. Wang, R. Liang, K-Y. Wang and H-C. Zhou. *Development of the design and synthesis of metal-organic frameworks (MOFs) - from large scale attempts, functional oriented modifications, to artificial intelligence (AI) predictions*. Chem. Soc. Rev. **2025**, 54, 367-395

[3] K. Guesh, C.A.D. Caiuby, Á. Mayoral, M. Díaz-García, I. Díaz, M. Sanchez-Sanchez, *Sustainable Preparation of MIL-100(Fe) and Its Photocatalytic Behavior in the Degradation of Methyl Orange in Water*. Cryst. Growth Des. **2017**, 17 (4), 1806–1813

[4] Z. Zheng, H. L. Nguyen, N. Hanikel, K. K.-Y. Li, Z. Zhou, T. Ma and O. M. Yaghi. *High-yield, green and scalable methods for producing MOF-303 for water harvesting from desert air*. Nat. Protoc. **2023**, 18, 136–156

[5] B. P. Carpenter, A. R. Talosig, B. Rose, G. D. Palma and J. P. Patterson. *Understanding and controlling the nucleation and growth of metal–organic frameworks.* Chem. Soc. Rev. **2023**, 52, 6918–6937

[6] Y. Guo, Z. Han, H. Min, Z. Chen, T. Sun, L. Wang, W. Shi and P. Cheng. *Bilanthanide Metal–Organic Frameworks for Instant Detection of 17β-Estradiol, a Vital Physiological Index*. Small Struct. **2022**, 3, 2100113

[7] P. T. Phan, J. Hong, N. Tran and T. H. Le. *The Properties of Microwave-Assisted Synthesis of Metal–Organic Frameworks and Their Applications*. Nanomaterials **2023**, 13, 352

[8] N. Stock and S. Biswas. *Synthesis of Metal-Organic Frameworks (MOFs): Routes to Various MOF Topologies, Morphologies, and Composites*. Chem. Rev. **2012**, 112, 933–969

[9] S. H. Jhung, J.-H. Lee, J. W. Yoon, C. Serre, G. Férey and J.-S. Chang. *Microwave synthesis of chromium terephthalate MIL-101 and its benzene sorption ability*. Adv. Mater. **2007**, 19, 121–124

[10] E. V. Perez, K. J. Balkus Jr., J. P. Ferraris and I. H. Musselman. *Mixed-matrix membranes containing MOF-5 for gas separations*. J. Membr. Sci. **2009**, 328, 165–173

[11] R. Ameloot, L. Stappers, J. Fransaer, L. Alaerts, B. F. Sels and D. E. De Vos. *Patterned Growth of Metal-Organic Framework Coatings by Electrochemical Synthesis*. Chem. Mater. **2009**, 21, 2580–2582

[12] A. Pichon, A. Lazuen-Garay and S. L. James. *Solvent-free synthesis of a microporous metal–organic framework*. CrystEngComm **2006**, 8, 211–214 RSC

[13] T. Friscic, D. G. Reid, I. Halasz, R. S. Stein, R. E. Dinnebier and M. J. Duer. *Ion- and liquid-assisted grinding: improved mechanochemical synthesis of metal-organic frameworks reveals salt inclusion and anion templating*. Angew. Chem., Int. Ed. **2010**, 49, 712–715

[14] W. Yuan, T. Friscic, D. Apperley and S. L. James. *High Reactivity of Metal–Organic Frameworks under Grinding Conditions: Parallels with Organic Molecular Materials*. Angew. Chem., Int. Ed. **2010**, 49, 3916–3919

[15] R. Zhang, C.-A. Tao, R. Chen, L. Wu, X. Zou and J. Wang. *Ultrafast Synthesis of Ni-MOF in One Minute by Ball Milling*. Nanomaterials **2018**, 8, 1067

[16] N. A. Khan and S. H. Jhung. *Synthesis of metal-organic frameworks (MOFs) with microwave or ultrasound: Rapid reaction, phase-selectivity, and size reduction*. Coord. Chem. Rev. **2015**, 285, 11–23

[17] J. H. Bang and K. S. Suslick. *Applications of Ultrasound to the Synthesis of Nanostructured Materials*. Adv. Mater. **2010**, 22, 1039–1059

[18] S. Hajra, M. Sahu, A. M. Padhan, I. S. Lee, D. K. Yi, P. Alagarsamy, S. S. Nanda and H. J. Kim. *A Green Metal–Organic Framework-Cyclodextrin MOF: A Novel Multifunctional Material Based Triboelectric Nanogenerator for Highly Efficient Mechanical Energy Harvesting*. Adv. Funct. Mater. **2021**, 31, 2101829

[19] V. Cabras, M. Pilloni, A. Scano, R. Lai, M.C. Aragoni, S.J. Coles, G. Ennas. *Mechanochemical Reactivity of Square-Planar Nickel Complexes and Pyridyl-Based Spacers for the Solid-State Preparation of Coordination Polymers: The Case of Nickel Diethyldithiophosphate and 4,4'-Bipyridine*. Eur. J. Inorg. Chem. **2017**, 13, 1908-1914

[20] M. Pilloni, F. Padella, G. Ennas, S. Lai, M. Bellusci, E. Rombi, F. Sini, M. Pentimalli, C. Delitala, A. Scano, et al. *Liquid-assisted mechanochemical synthesis of an Iron Carboxylate Metal Organic Framework and its evaluation in diesel fuel desulfurization*. Micropor. Mesopor. Mater. **2015**, 213, 14–21

[21] E. Colacino, G. Ennas, I. Halaz, A. Porcheddu, A. Scano. *Mechanochemistry: from soft matter to hard materials. An Introducion and a Practical Guide*, In: De Gruyter, ed.; **2021**.
